# A Geospatial Analysis of the Effects of Aviation Gasoline on Childhood Blood Lead Levels

**DOI:** 10.1289/ehp.1003231

**Published:** 2011-07-13

**Authors:** Marie Lynn Miranda, Rebecca Anthopolos, Douglas Hastings

**Affiliations:** Children’s Environmental Health Initiative, Nicholas School of the Environment, Duke University, Durham, North Carolina, USA

**Keywords:** avgas, aviation gasoline, blood lead, childhood, geospatial, lead poisoning

## Abstract

Background: Aviation gasoline, commonly referred to as avgas, is a leaded fuel used in small aircraft. Recent concern about the effects of lead emissions from planes has motivated the U.S. Environmental Protection to consider regulating leaded avgas.

Objective: In this study we investigated the relationship between lead from avgas and blood lead levels in children living in six counties in North Carolina.

Methods: We used geographic information systems to approximate areas surrounding airports in which lead from avgas may be present in elevated concentrations in air and may also be deposited to soil. We then used regression analysis to examine the relationship between residential proximity to airports and North Carolina blood lead surveillance data in children 9 months to 7 years of age while controlling for factors including age of housing, socioeconomic characteristics, and seasonality.

Results: Our results suggest that children living within 500 m of an airport at which planes use leaded avgas have higher blood lead levels than other children. This apparent effect of avgas on blood lead levels was evident also among children living within 1,000 m of airports. The estimated effect on blood lead levels exhibited a monotonically decreasing dose–response pattern, with the largest impact on children living within 500 m.

Conclusions: We estimated a significant association between potential exposure to lead emissions from avgas and blood lead levels in children. Although the estimated increase was not especially large, the results of this study are nonetheless directly relevant to the policy debate surrounding the regulation of leaded avgas.

Lead poisoning in children living in the United States has declined dramatically over the last several decades as a result of banning leaded gasoline, lead-based paint, and lead solder in plumbing. Nevertheless, children in the United States continue to be exposed to lead. The 2007–2008 National Health and Nutrition Examination Survey survey found blood lead levels at or above the Centers for Disease Control and Prevention (CDC) blood lead action level of 10 µg/dL in about 1.1% of 1- to 5-year-olds, or about 270,000 children (National Center for Health Statistics 2010). Even more worrisome is a large body of recent research that demonstrates negative health effects, including learning disabilities and behavioral disorders, associated with lead exposure levels well below the CDC action level (Canfield et al. 2003; Chiodo et al. 2004; Lanphear et al. 2000; Schnaas et al. 2006). A study by Miranda et al. (2007, 2009, 2010) suggests that early childhood blood lead levels as low as 2 µg/dL can have significant impacts on academic performance as measured by end-of-grade test scores. In response to this body of research, the CDC has stated that there is no safe level for blood lead in children (CDC 2005).

One source of lead exposure that is often overlooked is aviation fuel. Lead emitted from aircraft using leaded aviation gasoline (avgas) is currently the largest source of lead in air in the United States, constituting about 50% of lead emissions in the 2005 National Emissions Inventory [U.S. Environmental Protection Agency (EPA) 2010]. Although leaded gasoline for automobiles was phased out of use in the United States by 1995, lead is still permitted in avgas. Lead is added to avgas to achieve the high octane required for the engines of piston-driven airplanes. The most commonly used fuel for piston-driven aircraft in the United States is known as Avgas 100LL. Although the “LL” stands for low lead, 100LL gasoline contains up to 0.56 g/L lead (Royal Dutch Shell 2010). Another grade of avgas, Avgas 100, contains higher amounts of lead and is still in widespread use. Newer varieties of avgas without lead, including 82 UL and 94 UL, have been introduced recently. These unleaded fuels are not used as commonly as the two leaded grades, however, because their octane ratings are too low for many small aircraft engines.

Previous research indicates that lead levels in air near airports where planes use avgas are significantly higher than background levels. A study at the Santa Monica airport in California found that the highest lead levels occur close to airport runways and decrease exponentially with distance from an airport, dropping to background levels at about 1 km (U.S. EPA 2010). Another study at Toronto-Buttonville (Canada) airport found that the average air lead level near the airport was 4.2 times higher than the background air lead level in Toronto over a 24-hr period (Environment Canada 2000), and a study at Chicago (IL) O’Hare airport found that air lead levels were significantly higher downwind from the airport than upwind (Illinois Environmental Protection Agency 2002).

Thus, the combustion of leaded avgas by small airplane engines may pose a health risk to children who live or attend school near airports. The lead in air surrounding airports can be inhaled directly, or the lead may be ingested by children after it settles into soil or dust (U.S. EPA 2010). The U.S. EPA estimates that people living within 1 km of airports are at risk of being exposed to lead from avgas (Hitchings 2010). The U.S. EPA further notes that about 16 million people live within 1 km of an airport with planes using avgas, and 3 million children attend school within 1 km of these airports (U.S. EPA 2010).

Because of the risk of lead poisoning from avgas, environmental groups have pressured the U.S. EPA to take action to reduce lead emissions from aviation fuel. One environmental group, Friends of the Earth, has petitioned the U.S. EPA to find endangerment from and regulate lead in avgas. The U.S. EPA has responded with an Advanced Notice for Proposed Rulemaking on aviation fuel and solicited comments and further research about the effects of lead in avgas away (U.S. EPA 2010). The U.S. EPA has refrained from establishing a date by which aircraft would be required to use unleaded fuel [AOPA (Aircraft Owners and Pilots Association) ePublishing staff 2010].

Here we seek to contribute to research regarding the risk of lead in avgas by determining whether living near airports where avgas is used has a discernible impact on blood lead levels in children. Previous studies have examined whether lead from avgas is present in air and soil near airports. Our work seeks to link avgas exposure to childhood blood lead levels. To elucidate the effects of avgas on blood lead levels, we compared blood lead levels in children living near airports in six counties in North Carolina with those in children living farther away from airports but residing in the same counties. We used a multiple regression model to control for other variables that have previously been found to affect blood lead levels (CDC 1991, 1997; Sargent et al. 1995) in an effort to isolate the impact of avgas. The results of this study are directly relevant to the policy debate surrounding the regulation of leaded avgas.

## Methods

**Figure 1:**
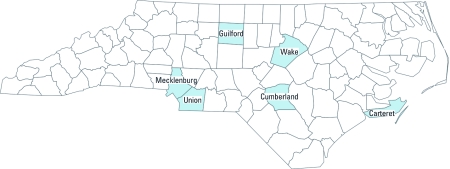
Study counties.

We obtained a database of airports in North Carolina from the U.S. EPA Office of Transportation and Air Quality (2008). The database contained estimates for the annual lead emissions from each airport, along with the spatial location of each facility. We used ArcGIS 9.3 (ESRI, Redlands, WA) to plot the locations of these airports against a county boundary map of North Carolina. We selected six counties in North Carolina (Carteret, Cumberland, Guilford, Mecklenburg, Union, and Wake) (Figure 1). Counties were selected based on whether they contained multiple airports with significant air traffic, where significant numbers of children had been screened for lead exposure, and where the county tax assessor data would allow us to control for age of housing as an important confounder when assessing avgas as a source of lead exposure (Table 1). Because we wanted to control for risk from deteriorating lead-based paint, we selected counties where the county tax assessor data contained a well-populated field for age of housing. We obtained North Carolina blood lead surveillance data for all children in the study counties between the ages of 9 months and 7 years who had been tested for lead between 1995 and 2003 from the Children’s Environmental Health Branch within the North Carolina Department of Environment and Natural Resources (North Carolina Childhood Lead Poisoning Prevention Program 2004). Because we were unable to ascertain where the children attended school, we were not able to control for the location of their school relative to the airports. Most of the children screened for lead are not yet old enough to be attending school. All aspects of this study were conducted in accordance with a human subjects research protocol approved by the institutional review board (IRB) of Duke University.

**Table 1:** Number of airports, estimate of lead emissions from aircrafts, and number of blood lead screens among children 9 months to 7 years of age in study counties, North Carolina (1995–2003).

County	No. of airports	Estimated lead emissions (tons/year)	No. of blood lead screens
Carteret		8		0.224		3,333
Cumberland		11		0.238		14,854
Guilford		10		0.369		27,043
Mecklenburg		10		0.894		47,510
Union		14		0.285		3,387
Wake		13		0.624		29,070

**Figure 2:**
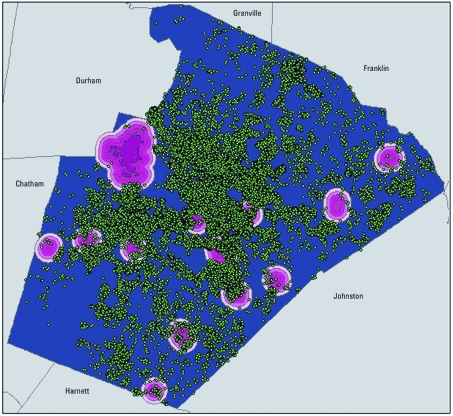
Airports buffered at distances of 500 m, 1,000 m, 1,500 m, and 2,000 m in Wake County, North Carolina, plotted along with a jittered representation of the residential addresses of the children screened for blood lead.

After selecting our six study counties, we used geographic information systems (GIS) to delineate fixed distance areas around each airport where aircraft use avgas. We also used GIS to connect the point locations of the airports given by address to tax parcel layers for each county via shared geography. The tax parcel layers contain a polygon shape representing the property boundary of each airport. We then created buffers around each of the airport polygons to represent the area in which airplane emissions could affect air lead levels. Because previous research has indicated that lead concentrations increase exponentially with proximity to airports (Piazza 1999), we created buffers that extended 500 m, 1,000 m, 1,500 m, and 2,000 m from the polygon edges of the airport tax parcels. Figure 2 depicts this approach using the example of Wake County. Airports are indicated by the darkest shade of pink, with the different distance buffers represented by increasingly lighter shades of pink. The residential addresses of the children who were screened for blood lead is then overlaid, as shown by the green points. In accordance with our IRB protocol, the green dots do not represent the actual locations where children were screened for lead. For publicly displayed maps like Figure 2, we randomly move the actual location of the child within a fixed radial buffer, a technique known as jittering. The analysis itself, however, is done on the true locations of the children. The 500-m, 1,000-m, 1,500-m, and 2,000-m buffers only approximate the area that could be affected by lead emissions from airports, as wind directions can alter the dispersal pattern of lead particles. Nevertheless, with varied wind directions and planes that take off in multiple directions, our buffers offer a reasonable approximation of the area over which lead from avgas might disperse.

North Carolina maintains a mandatory statewide registry of blood lead surveillance data. We obtained North Carolina blood lead surveillance data for 1995–2003 (North Carolina Childhood Lead Poisoning Prevention Program 2004), because these years bracket the 2000 census data. In previous work designed to develop childhood lead exposure risk models (Kim et al. 2008; Miranda et al. 2002), we had already geocoded the residential addresses of children screened for lead. Our geocoding success rates ranged from 37 to 89% across the six study counties. Details on how the blood lead surveillance data were processed are described by Miranda et al. (2002) and Kim et al. (2008).

We then joined the buffered airport polygons in our six study counties with the geocoded addresses of children who have been screened for blood lead. This enabled us to generate a table containing blood lead screening results and four dummy variables representing whether each child lived within 500 m, 1,000 m, 1,500 m, or 2,000 m of an airport.

We supplemented the blood lead screening and airport location data with data from county tax assessor databases on age of housing (to control for lead exposure risks from deteriorating lead-based paint), resolved at the individual tax parcel level. In addition, we used U.S. Census 2000 data on household median income (measured in tens of thousands) and proportion receiving public assistance, which were obtained at the census block group level (U.S. Census Bureau 2002), as well as proportion non-Hispanic black and proportion Hispanic, which were obtained at the census block level (U.S. Census Bureau 2001). Because previous work has shown the season of blood lead screening to be a significant predictor of blood lead levels (i.e., warm months are correlated with higher lead exposure from lead-based paint) (Johnson et al. 1996; Kim et al. 2008; Miranda et al. 2007; Yiin et al. 2000), we created individual level dummy variables representing the season in which each child was screened for lead. Because the blood lead screening data are right-skewed, we used the natural logarithm of blood lead level in our analyses. We used the spatial data architecture described above to regress logged blood lead levels on the proximity to airport variable, controlling for age of housing, season in which the child was screened, and the census demographic variables. We used multivariable regression analysis clustered at the census block group level with inverse population weights at the tax parcel level to ensure that parcels with multiple blood lead screens did not overly influence the analysis. We implemented crude and adjusted regression models for each of the four proximity to airport variables. We used a categorical distance to airport variable with 0–500 m, 501–1,000 m, 1,001–1,500 m, and 1,501–2,000 m, with a reference group of > 2,000 m. In addition, we performed a sensitivity analysis on our findings. First, we investigated whether the use of inverse population weights accounted for possible correlation among observations from the same tax parcel by running multilevel random intercept models designating the parcel as the grouping variable. Second, we considered the possibility of temporal confounding by including the lead screen year as a factor in each model with the reference year as 1995. Results regarding the importance of distance to airports were robust across these alternative specifications. We examined the results of these regressions to determine whether living near an airport using avgas had significant effects on blood lead levels. Statistical significance was set at α = 0.05

## Results

Blood lead screening data were available for 125,197 children in the study counties (Table 1), including 13,478 children living within 2,000 m of an airport polygon in the six study counties (Table 2).

**Table 2:** Individual and group-level characteristics of children 9 months to 7 years of age who were screened for blood lead 1995–2003 (*n* = 125,197).

Characteristic	Value
Individual level		
Blood lead level [µg/dL (mean ± SD)]		3.88 ± 2.94
Season in which blood lead screening occurred [*n* (%)]*a*		
Winter		27,189 (21.72)
Spring		30,593 (24.44)
Summer		35,256 (28.16)
Fall		32,159 (25.69)
Residential proximity to airport [*n* (%)]		
Within 500 m		1,267 (1.01)
Within 1,000 m		3,649 (2.92)
Within 1,500 m		8,122 (6.49)
Within 2,000 m		13,478 (10.77)
> 2,000 m		111,719 (89.23)
Year residence of child built (mean ± SD)		1970 ± 20.10
Group level (mean ± SD)		
Proportion black*b*		0.39 ± 0.33
Proportion Hispanic*b*		0.09 ± 0.15
Household median income ($10,000s)*c*		4.38 ± 2.09
Proportion receiving public assistance*c*		0.04 ± 0.05
**a**Winter: December–February; spring: March–May; summer: June–August; fall: September–November. **b**Resolved at the census block level. **c**Resolved at the census block group level.		

Our statistical results are shown in Table 3. In unadjusted models, logged blood lead levels were significantly and positively associated with residential proximity to an airport, with the size of the association being larger for children living closer to airports. Although controlling for individual- and group-level confounders attenuated the association between logged blood lead levels and residential proximity to an airport, evidence of a deleterious relationship remained. In the adjusted models, control variables behaved as expected: Relative to being screened in the winter season, children tested in the spring, summer, or fall had increased blood lead levels, on average. Residence in poor and minority neighborhoods was also associated with elevated lead levels. In contrast, recently constructed housing units were associated with decreased mean lead levels. The above associations were consistent between the within-distance and categorical distance regression models.

**Table 3:** Change in logged blood lead level associated with a child’s residential proximity to airport using multiple linear regression (*n* = 125,197).

Within distance buffers*a*				Categorical distance measure			
Covariate	Coefficient (95% CI)		Covariate	Coefficient (95% CI)	
Unadjusted								
Within 500 m		0.089	(0.034 to 0.144)^#^		Between 0 and 500 m		0.094	(0.038 to 0.150)^#^
Within 1,000 m		0.084	(0.036 to 0.133)^#^		Between 501 and 1,000 m		0.085	(0.027 to 0.142)^#^
Within 1,500 m		0.077	(0.039 to 0.116)^#^		Between 1,001 and 1,500 m		0.071	(0.023 to 0.119)^#^
Within 2,000 m		0.052	(0.018 to 0.087)^#^		Between 1,501 and 2,000 m		0.016	(–0.022 to 0.053)
					> 2,000 m		Reference	
Adjusted*b*								
Within 500 m		0.043	(0.006 to 0.080)**		Between 0 and 500 m		0.043	(0.006 to 0.080)**
Within 1,000 m		0.037	(0.010 to 0.065)^#^		Between 501 and 1000 m		0.034	(–0.003 to 0.072)*
Within 1,500 m		0.021	(0.0008 to 0.041)**		Between 1,001 and 1,500 m		0.007	(–0.020 to 0.034)
Within 2,000 m		0.003	(–0.013 to 0.020)		Between 1,501 and 2,000 m		–0.019	(–0.041 to 0.003)*
					> 2,000 m		Reference	
**a**Within-distance thresholds were entered in separate regression models. **b**Adjusted models control for census block level proportion black and proportion Hispanic, census block group level percent population receiving public assistance and household median income, as well as individual level dummy variables for the season in which a child was screened for blood lead. *p < 0.10. **p < 0.05. ^#^*p* < 0.01.								

In the within-distance buffer specification for the adjusted models, blood lead levels were significantly associated with residing within 500 m [coefficient = 0.043; 95% confidence interval (CI), 0.006–0.080]; 1,000 m (coefficient = 0.037; 95% CI, 0.010–0.065), and 1,500 m (coefficient = 0.021; 95% CI, 0.0008–0.041) of an airport. Blood lead levels were not associated with living at greater distances. Importantly, the magnitude of the coefficient on the distance to airport variables was largest for those children living within 500 m and decreased in a dose–response fashion out to 1,500 m. On the basis of distance to airport coefficients, children living within 500 m, 1,000 m, or 1,500 m of an airport had average blood lead levels that were 4.4, 3.8, or 2.1% higher, respectively, than other children.

In the categorical distance specification, compared with the reference category (> 2,000 m from an airport), children living within 500 m of an airport had blood lead levels that were, on average, 4.4% higher (coefficient = 0.043; 95% CI, 0.006–0.080) (Table 3). In addition, the coefficient for the 501–1000 m category was marginally significant (coefficient = 0.034; 95% CI, –0.003 to 0.072). Neither the 1,001–1,500 m nor the 1,501–2,000 m category was significant at the 5% level, with coefficient estimates near the null value. These results taken collectively suggest that children living within 500 m and within 1,000 m are driving the results in the models that entered the within-distance threshold variables separately.

## Discussion

Based on the geospatial and statistical analysis presented above, lead from avgas may have a small (2.1–4.4%) but significant impact on blood lead levels in children who live in proximity to airports where avgas is used. The magnitude of the estimated effect of living near airports was largest for those children living within 500 m and decreased in a monotonic fashion out to 1,500 m. Because our model takes into account only whether a child is living anywhere in a fixed distance (500 m, 1,000 m, or 1,500 m) radius of an airport, children who live very close to or downwind from a runway could be affected more significantly than the average value that we estimate for all children living within the buffer.

Our finding that living beyond 1,000 m of an airport using avgas does not have a significant relationship with blood lead levels is reasonably consistent with previous research suggesting that lead drops to background levels beyond 1,000 m from an airport (Piazza 1999).

Our study has several important limitations. It does not take into account wind patterns that could increase the extent of the area containing lead particles from avgas in certain directions and decrease it in others. Furthermore, our model considers only whether children live anywhere within a particular distance from an airport and does not consider the fact that some points within this area could have higher air lead concentrations than others. Our modeling of the relationship between avgas and blood lead could be improved by incorporating wind direction information, by obtaining information about where piston-engine aircraft typically take off or land at each airport, and by controlling for air traffic volume. In addition, the variability in our geocoding success rates may introduce spatial bias. To partially address this, we re-ran the analysis without Union County, which had the lowest geocoding rate (37% compared with 58% for the remaining counties combined). The distance from airport results were robust to this change in the data set. We also note that if one includes a rural county like Union County, geocoding rates are inevitably poor. We felt it important to include a rural county, so we reported results with Union County data. Nonetheless, the analysis presented here would be strengthened with better geocoding rates. Finally, extending the study to additional counties throughout the United States could increase sample size and determine whether the trends that we observed in North Carolina are replicated elsewhere in the country. The methods we describe here for constructing buffer zones around airports could easily be replicated in other areas nationally (or internationally).

## Conclusions

Our analysis indicates that living within 1,000 m of an airport where avgas is used may have a significant effect on blood lead levels in children. Our results further suggest that the impacts of avgas are highest among those children living closest to the airport. This study adds to the literature examining whether leaded avgas poses risks to children’s health and speaks directly to the ongoing policy debate regarding the regulation of leaded avgas.

## References

[r1] AOPA (Aircraft Owners and Pilots Association) ePublishing staff (2010). EPA confirms: No lead ban deadline looms on avgas.. http://www.aopa.org/advocacy/articles/2010/100728avgas_epa.html.

[r2] Canfield RL, Henderson CR, Cory-Slechta DA, Cox C, Jusko TA, Lanphear BP (2003). Intellectual impairment in children with blood lead concentrations below 10 microg per deciliter.. N Engl J Med.

[r3] CDC (Centers for Disease Control and Prevention) (1991). Preventing Lead Poisoning in Young Children: A Statement by the Centers for Disease Control.

[r4] CDC (Centers for Disease Control and Prevention) (1997). Screening Young Children for lead Poisoning: Guidance for State and Local Public Health Officials.

[r5] CDC (Centers for Disease Control and Prevention) (2005). Preventing Lead Poisoning in Young Children.

[r6] Chiodo LM, Jacobson SW, Jacobson JL (2004). Neurodevelopmental effects of postnatal lead exposure at very low levels.. Neurotoxicol Teratol.

[r7] Environment Canada (2000). Airborne Paniculate Matter, Lead and Manganese at Buttonville Airport.. CEP Project.

[r8] Hitchings M. (2010). U.S. EPA aims to slash aviation gasoline emissions.. Glob Refining Fuels Today.

[r9] Illinois Environmental Protection Agency BoA (2002). Final Report: Chicago O’Hare Airport Air Toxic Monitoring Program June–December, 2000.. http://www.epa.state.il.us/air/ohare/ohare-toxic-report.pdf.

[r10] Johnson DL, McDade K, Griffith DA (1996). Seasonal variation in paediatric blood lead levels in Syracuse, NY, USA.. Environ Geochem.

[r11] Kim D, Galeano MA, Hull A, Miranda ML (2008). A framework for widespread replication of a highly spatially resolved childhood lead exposure risk model.. Environ Health Perspect.

[r12] Lanphear BP, Dietrich K, Auinger P, Cox C (2000). Cognitive deficits associated with blood lead concentrations < 10 µg/dL in US children and adolescents.. Public Health Rep.

[r13] Miranda ML, Dolinoy DC, Overstreet MA (2002). Mapping for prevention: GIS models for directing childhood lead poisoning prevention programs.. Environ Health Perspect.

[r14] Miranda ML, Kim D, Overstreet Galeano MA, Paul C, Hull A, Morgan SP (2007). The relationship between early childhood blood lead levels and performance on end of grade tests.. Environ Health Perspect.

[r15] Miranda ML, Kim D, Reiter J, Overstreet Galeano MA, Maxson P (2009). Environmental contributors to the achievement gap.. Neurotoxicology.

[r16] Miranda ML, Maxson P, Kim D (2010). Early childhood lead exposure and exceptionality designations for students.. Int J Child Health Hum Dev.

[r17] National Center for Health Statistics (2010). National Health and Nutrition Examination Survey Data.

[r18] North Carolina Childhood Lead Poisoning Prevention Program (2004). North Carolina Childhood Lead Screening Data.

[r19] Piazza B (1999). Santa Monica Municipal Airport: A Report on the Generation and Downwind Extent of Emissions Generated from Aircraft and Ground Support Operations.. http://yosemite.epa.gov/oar/communityassessment.nsf/6ce396ab3fa98ee485256db0004acd94/$FILE/Santa_Monica.pdf.

[r20] Royal Dutch Shell (2010). Avgas Grades and Specifications.. http://www.shell.com/home/content/aviation/products/fuels/types/avgas/.

[r21] Sargent JD, Brown MJ, Freeman JL, Bailey A, Goodman D, Freeman DH (1995). Childhood lead poisoning in Massachusetts communities: its association with sociodemographic and housing characteristics.. Am J Public Health.

[r22] Schnaas L, Rothenberg SJ, Flores MF, Martinez S, Hernandez C, Osorio E (2006). Reduced intellectual development in children with prenatal lead exposure.. Environ Health Perspect.

[r23] U.S. Census Bureau (2001). Census 2000 Summary File 1. North Carolina.. http://factfinder.census.gov/servlet/DTSubjectShowTablesServlet?_ts=333454048306.

[r24] U.S. Census Bureau (2002). Census 2000 Summary File 3.. http://factfinder.census.gov/servlet/DTSubjectShowTablesServlet?_ts=333451408273.

[r25] U.S. EPA (U.S. Environmental Protection Agency) (2008). Estimated Pb Emissions from All NC Airport Facilities.

[r26] U.S. EPA (U.S. Environmental Protection Agency) (2010). Advance notice of proposed rulemaking on lead emissions from piston-engine aircraft using leaded aviation gasoline.. Fed Regist.

[r27] Yiin LM, Rhoads GG, Lioy PJ (2000). Seasonal influences on childhood lead exposure.. Environ Health Perspect.

